# Airway obstruction and bronchial reactivity from age 1 month until 13 years in children with asthma: A prospective birth cohort study

**DOI:** 10.1371/journal.pmed.1002722

**Published:** 2019-01-08

**Authors:** Henrik Wegener Hallas, Bo Lund Chawes, Morten Arendt Rasmussen, Lambang Arianto, Jakob Stokholm, Klaus Bønnelykke, Hans Bisgaard

**Affiliations:** Copenhagen Prospective Studies on Asthma in Childhood, Herlev and Gentofte Hospital, University of Copenhagen, Copenhagen, Denmark; Edinburgh University, UNITED KINGDOM

## Abstract

**Background:**

Studies have shown that airway obstruction and increased bronchial reactivity are present in early life in children developing asthma, which challenges the dogma that airway inflammation leads to low lung function. Further studies are needed to explore whether low lung function and bronchial hyperreactivity are inherent traits increasing the risk of developing airway inflammation and asthmatic symptoms in order to establish timely primary preventive initiatives.

**Methods and findings:**

We investigated 367 (89%) of the 411 children from the at-risk Copenhagen Prospective Studies on Asthma in Childhood 2000 (COPSAC_2000_) birth cohort born to mothers with asthma, who were assessed by spirometry and bronchial reactivity to methacholine from age 1 month, plethysmography and bronchial reversibility from age 3 years, cold dry air hyperventilation from age 4 years, and exercise challenge at age 7 years. The COPSAC pediatricians diagnosed and treated asthma based on symptom load, response to inhaled corticosteroid, and relapse after treatment withdrawal according to a standardized algorithm. Repeated measures mixed models were applied to analyze lung function trajectories in children with asthma ever or never at age 1 month to 13 years. The number of children ever versus never developing asthma in their first 13 years of life was 97 (27%) versus 270 (73%), respectively. Median age at diagnosis was 2.0 years (IQR 1.2–5.7), and median remission age was 6.2 years (IQR 4.2–7.8). Children with versus without asthma had reduced lung function (*z-*score difference, forced expiratory volume, −0.31 [95% CI −0.47; −0.15], *p <* 0.001), increased airway resistance (*z-*score difference, specific airway resistance, +0.40 [95% CI +0.24; +0.56], *p <* 0.001), increased bronchial reversibility (difference in change in forced expiratory volume in the first second [ΔFEV_1_], +3% [95% CI +2%; +4%], *p <* 0.001), increased reactivity to methacholine (*z-*score difference for provocative dose, −0.40 [95% CI −0.58; −0.22], *p <* 0.001), decreased forced expiratory volume at cold dry air challenge (ΔFEV_1_, −4% [95% CI −7%; −1%], *p <* 0.01), and decreased forced expiratory volume after exercise (ΔFEV_1_, −4% [95% CI −7%; −1%], *p =* 0.02). Both airway obstruction and bronchial hyperreactivity were present before symptom debut, independent of disease duration, and did not improve with symptom remission. The generalizability of these findings may be limited by the high-risk nature of the cohort (all mothers had a diagnosis of asthma), the modest study size, and limited ethnic variation.

**Conclusions:**

Children with asthma at some point at age 1 month to 13 years had airway obstruction and bronchial hyperreactivity before symptom debut, which did not worsen with increased asthma symptom duration or attenuate with remission. This suggests that airway obstruction and bronchial hyperreactivity are stable traits of childhood asthma since neonatal life, implying that symptomatic disease may in part be a consequence of these traits but not their cause.

## Abstract 2

Hans Bisgaard and colleagues analyze cohort data for evidence as to whether symptomatic asthma is the cause or consequence of early-life airway obstruction and bronchial hyperreactivity.

## Author summary

**Why was this study done?:**

- It is believed that asthma develops from inflammation in the lungs that leads to loss of lung function, but low lung function may be an inherent trait in children at risk of asthma instead of a consequence of inflammation.
- It is important to explore whether low lung function is an inherent trait that increases the risk of developing airway inflammation and asthma in order to establish primary preventive initiatives for low lung function.

**What did the researchers do and find?:**

- Ninety-seven children who developed asthma and 270 children without asthma from the Danish COPSAC_2000_ birth cohort born to mothers with asthma were studied extensively with longitudinal lung function measurements from age 1 month to 13 years.
- Lung function was measured by spirometry from age 1 month and plethysmography from age 3 years, including assessments of bronchial reversibility to inhaled β2-agonist from age 3 years. Bronchial reactivity was assessed by methacholine challenge from age 1 month, cold dry air hyperventilation from age 4 years, and exercise challenge at age 7 years.
- Children developing asthma had reduced lung function from age 1 month throughout childhood compared to the children without asthma.
- The lung function deficit was present before the children developed asthma, did not progress with symptoms, and remained even if symptoms ceased.

**What do these findings mean?:**

- Low lung function appears to begin in early childhood in a group of children who will develop asthma. The lung function trait is established prior to development of airway inflammation and asthma, and does not worsen with increased duration of asthma symptoms. As airway obstruction and increased bronchial reactivity manifest as early as 1 month of age, it is possible that preventive measures undertaken during pregnancy will be most effective.

## Introduction

The current paradigm of asthma pathophysiology suggests that an inflammatory process in the airways leads to progressive bronchial hyperreactivity and lung function deficits [1]. An alternative hypothesis is causality in the opposite direction, i.e., that reduced airway caliber and bronchial hyperreactivity are inherent and stable traits that increase the risk of asthmatic symptoms, exaggerated hyperreactivity, and intermittent airway obstruction from a superimposed inflammatory process. In support of this, anti-inflammatory inhaled corticosteroid (ICS) treatment does not affect the natural course of lung function in children [2–6]. Furthermore, reticular basement membrane and airway smooth muscle thickness are unrelated to inflammatory cell counts in bronchial biopsies [7]. This alternative causal direction from airway obstruction and bronchial hyperreactivity to asthma symptoms implies that prevention must take place before symptom debut and maybe even before birth [8,9].

We aimed to study this alternative paradigm with rigorous prospective assessment of disease duration and remission. Spirometry and bronchial reactivity to methacholine were assessed in neonates [10,11]. Neonatal spirometry was volume-anchored and therefore comparable to repeated assessments of spirometry and bronchial reactivity during childhood [12–15]. Effort-independent measures of airway resistance and measures of bronchial reactivity were recorded until age 13 years.

## Methods

This study is reported as per the Strengthening the Reporting of Observational Studies in Epidemiology (STROBE) guidelines (S1 STROBE Checklist).

The study was nested in Copenhagen Prospective Studies on Asthma in Childhood 2000 (COPSAC_2000_): a Danish prospective birth cohort study of 411 infants born during 1998–2001 to mothers with a history of asthma [16]. At enrollment at age 1 month, we excluded any child with a history of symptoms of lower airway infection or neonatal mechanical ventilation, preterm birth (gestational age < 36 weeks), or any congenital abnormality or systemic illness. The children were examined at half-yearly scheduled visits until age 7 years, and again at age 13 years, including assessments of lung function 11 times during childhood. Thus, longitudinal lung function assessment during childhood was a strong and predefined focus of COPSAC_2000_, which is detailed in the cohort baseline paper [16], but there was no prospective analysis plan for how to model the data with respect to asthma development.

The local ethics committee (KF 01-289/96) and the Danish Data Protection Agency (2015-41-3696) approved the study. Both parents gave oral and written informed consent before enrollment.

### Lung function

#### Neonatal spirometry

Neonatal spirometry was performed at age 1 month by forced flow volume measurements applying the volume-anchored raised volume rapid thoracoabdominal compression technique [10,11]. Forced expiratory volume in the first 0.5 seconds (FEV_0.5_) and forced expiratory flow at 50% of expiration (FEF_50_) were calibrated for gestational age at birth, sex, height, and weight.

#### Spirometry

Spirometry was performed half-yearly between age 5 and 7 years and at 13 years using a MasterScope Pneumoscreen spirometer (Erich Jaeger, Würzburg, Germany) [17]. Forced expiratory volume in the first second (FEV_1_) and maximal mid-expiratory flow (MMEF) were calibrated for height, sex, and age.

To compare the neonatal FEV_0.5_ and FEF_50_ with FEV_1_ and MMEF data from later in childhood, the measurements were *z-*scored based on children never developing asthma and are subsequently referred to as FEV_z_ and MMEF_z_.

#### Whole body plethysmography

Whole body plethysmography was performed half-yearly between age 3 and 7 years and at 13 years using a MasterScreen sealed bodybox (Erich Jaeger, Würzburg, Germany). Measurements were obtained during tidal breathing using a mouthpiece [18]. Specific airway resistance (sRaw) was calibrated for height, sex, and age; logarithmically transformed to achieve normal distribution of the data; and subsequently *z-*scored (sRaw_z_).

### Bronchial reversibility

Bronchial reversibility was measured at age 3, 5, 7, and 13 years by whole body plethysmography and at 5, 7, and 13 years by spirometry as the relative change from baseline 15 minutes after inhalation of β2-agonist. At age 3–7 years, terbutaline, 500 μg (2 × 250 μg) pMDI (Bricanyl, AstraZeneca, Cambridge, UK) was delivered via a metal spacer (Nebuchamber, AstraZeneca, Cambridge, UK). At 13 years, we used salbutamol, 400 μg (2 × 200 μg) from a discus DPI (Ventoline, GlaxoSmithKline Pharma, Middlesex, UK).

### Bronchial reactivity

#### Methacholine challenge in neonates

Baseline FEV_0.5_ was measured after a saline inhalation and following subsequent inhalations of methacholine in quadrupling dose steps from 0.04 to 16.67 μmol/l delivered by a dosimeter attached to a nebulizer (SPIRA 08 TSM 133, Respiratory Care Center, Hämeenlinna, Finland) [10,11]. Bronchial reactivity was assessed from repeated measurements of FEV_0.5_ and continuous transcutaneous oxygen pressure (PtcO_2_) (TCM3, Radiometer, Copenhagen, Denmark) [10,11].

#### Methacholine challenge in school age children

Methacholine challenge in school-age children was performed at 7 and 13 years, measuring FEV_1_ after a saline inhalation and after subsequent inhalations of methacholine in quadrupling dose steps from 0.06 to 29.72 μmol/l (SPIRA 08 TSM 133, Respiratory Care Center, Hämeenlinna, Finland).

Bronchial reactivity to methacholine in neonates and school-age children was estimated from dose–response curves fitted with a logistic function as the provocative dose of methacholine producing a 15% decrease in PtcO_2_ (PD_15_) in the neonates, according to our previous sensitivity analysis in the cohort [11], and as the provocative dose of methacholine producing a 20% decrease in FEV_1_ (PD_20_) at age 7 and 13 years, according to the ATS/ERS guidelines [19]. To enable comparison of the assessments, PD_15_ and PD_20_ were logarithmically transformed to achieve normal distribution of the data, were *z-*scored, and are subsequently referred to as PD_z_.

#### Cold dry air challenge

Cold dry air (**−**18°C) was delivered by a respiratory heat exchange system (Erich Jaeger, Würzburg, Germany) with an animated computer program guiding the child to hyperventilate at a level corresponding to 1 l/min/kg bodyweight for 4 minutes using a facemask with a mouthpiece [20]. Bronchial reactivity was determined as the change from baseline in sRaw at 4 and 6 years and in FEV_1_ and MMEF at 6 years.

#### Exercise challenge

At 7 years, the child exercised on a motor-driven treadmill for at least 4 minutes with a pulse rate >80% of the maximal pulse [19] breathing fully dehumidified atmospheric air through a facemask (Hans Rudolph, Kansas City, MO, US). Spirometry was performed before, and 1, 3, 5, 10, and 15 minutes after exercise using the maximum percentage decline in FEV_1_ from baseline within 10 minutes after exercise as the outcome.

We excluded lung function measurements in children with respiratory tract symptoms in the preceding week and if β_2_-agonists had been used within the preceding 12 hours. Bronchial challenge test results were excluded if the child had a respiratory infection in the preceding 3 weeks [21].

### Monitoring of lung symptoms

The parents were taught to recognize troublesome lung symptoms including noisy breathing (wheezing or whistling sounds), breathlessness, shortness of breath, and persistent coughing at a session at the research unit. They subsequently recorded their child’s troublesome lung symptoms, i.e., symptoms significantly affecting the well-being of their child, in a daily diary as a composite score (yes/no) from age 1 month to 7 years. They were requested to bring the child to the COPSAC clinic at each episode of 3 consecutive days with symptoms for examination and verification of the symptoms by the COPSAC pediatricians [2,22]. If the child was diagnosed with asthma, the diary recording of lung symptoms was continued beyond age 7.

### Asthma diagnosis

#### Age 1 month–7 years

Asthma was diagnosed according to a predefined validated quantitative diary-based algorithm [22]. If the child experienced 5 episodes of troublesome lung symptoms within 6 months, daily symptoms for 4 weeks, or acute severe symptoms requiring hospitalization, high-dose ICS, or systemic corticosteroids, then a 3-month course of budesonide 200 μg bid (Spirocort, AstraZeneca, Cambridge, UK) was prescribed, and the child was examined by a chest X-ray and a sweat chloride test to exclude other chronic lung disorders. Asthma was diagnosed in children relapsing after ICS withdrawal within the following year, defined as either 2 episodes within 3 months or 2 consecutive weeks with symptoms, which resulted in a 6-month course of ICS and an additional 12-month course at subsequent relapses. Remission was defined as the child being without symptoms and treatment for 1 year.

#### After age 7 years

Children with troublesome lung symptoms after age 7 years were prescribed inhaled as-needed β2-agonist (terbutaline 250 μg; Bricanyl, AstraZeneca, Cambridge, UK). If β2-agonist was needed more than 2 days per week and not only in relation to scheduled physical activity, the child was prescribed a 3-month course of ICS (budesonide 400 μg/day [2 × 200 μg]; Spirocort, AstraZeneca, Cambridge, UK) and subsequently followed the same diagnostic procedure for asthma as described above. At the 13-year follow-up visit, the Danish National Medicine Registry was accessed and screened for any asthma drug prescriptions from age 7 to 13 years.

Only children attending the clinic at least once at age 0.5–4 years and at age 4–7 years or 13 years were included in the analyses.

### Allergen sensitization

Allergen sensitization towards 10 aeroallergens—birch, grass, mugwort, horse, dog, cat, *Dermatophagoides pteronyssinus*, *D*. *farinae*, *Cladosporium herbarum*, and *Alternaria alternata*—was determined at age 13 years by skin prick test [23] and by measuring blood levels of specific IgE [24]. Allergic sensitization was defined as any wheal > 3 mm and/or any specific IgE value ≥ 0.35 kU/A.

Description of baseline characteristics of the cohort, including pre-, peri- and postnatal factors is provided in S1 Text.

### Statistical analyses

Baseline characteristics of included versus excluded children were compared using Fisher’s exact test, Student’s *t* test, and Wilcoxon 2-sample test.

The relationship between development of FEV_z_, MMEF_z_, sRaw_z_, and PD_z_ in the first 13 years of life and asthma status was analyzed with mixed models taking repeated participant measurements into account. The statistic extracted from these mixed models was the fixed effect of group, i.e., asthma versus no asthma. To test the effect of the normalization of the lung function measurements, which was done to enable comparison of measurements conducted at varying ages, we also ran the analyses with raw, untransformed data and subsequently investigated the normal distribution of the residuals from the different models with Q-Q plots.

The cold dry air challenge by spirometry (6 years) and exercise challenge (7 years) were only performed once for each child, and the cross-sectional difference in these measures between children with asthma and without asthma was analyzed by Student’s *t* test comparison of means.

We subsequently investigated whether development of FEV_z_, MMEF_z_, sRaw_z_, and PD_z_ prior to onset of asthma was different from their development in children never developing asthma. This was done in mixed models comparing FEV_z_, MMEF_z_, sRaw_z_, and PD_z_ measurements obtained before diagnosis in children developing asthma with measurements in children never developing asthma, evaluating the fixed effect of group, i.e., asthma versus no asthma. As an ancillary analysis, we also investigated by *t* tests whether neonatal FEV_0.5_, FEF_50_, and PD_15_ were different in neonates who subsequently developed asthma versus neonates who did not develop asthma.

The effect of duration of asthma in the first 13 years of life on lung function development, i.e., whether lung function declined over time with increasing disease length, was investigated by calculating the duration of symptomatic disease preceding each lung function measurement. The mixed models used for this analysis were adjusted for time since remission and estimated the slope of the lung function during the period with asthma. The statistic extracted from these mixed models was the fixed effect of time, i.e., disease length in the observation period. To further investigate whether asthma status affected measures of FEV_z_, MMEF_z_, sRaw_z_, and PD_z_ over time, age at measurement was included in the analyses as an interaction term.

Finally, lung function development after remission of asthma was analyzed in mixed models by evaluating the change over time in FEV_z_, MMEF_z_, sRaw_z_, and PD_z_ after remission, evaluating the fixed effect of time since remission.

As post hoc analyses, we investigated development of FEV_z_, MMEF_z_, sRaw_z_, and PD_z_ in children with remission of asthma, i.e., early-transient symptoms, compared to children with persistent symptoms and children never developing asthma. We also investigated development of lung function in children with asthma who had specific allergen sensitization at age 13 year compared to children with asthma without sensitization. These post hoc analyses were done to investigate whether there were specific lung function trajectories in different wheeze phenotypes and whether development of atopy influenced the trajectories. The analyses were done with mixed models, evaluating the fixed effect of wheeze phenotype and sensitization status.

The effect of missing observations for longitudinal values for FEV_z_, MMEF_z_, sRaw_z_, and PD_z_ was investigated by multiple imputation (for details see S1 Text).

All analyses were performed in R with the package *lme4* (version 1.1.14) [25] for linear mixed models as well as the package *mice* (version 3.3.0). All estimates are presented with 95% confidence intervals; *p*-values < 0.05 were considered significant. The graphical presentations of the results were made with ggplot2 (version 3.0.0), and the longitudinal curves of FEV_z_, MMEF_z_, and sRaw_z_ development were smoothed with loess regression function.

The data are available in S1 Data.

## Results

**Fig 1:**
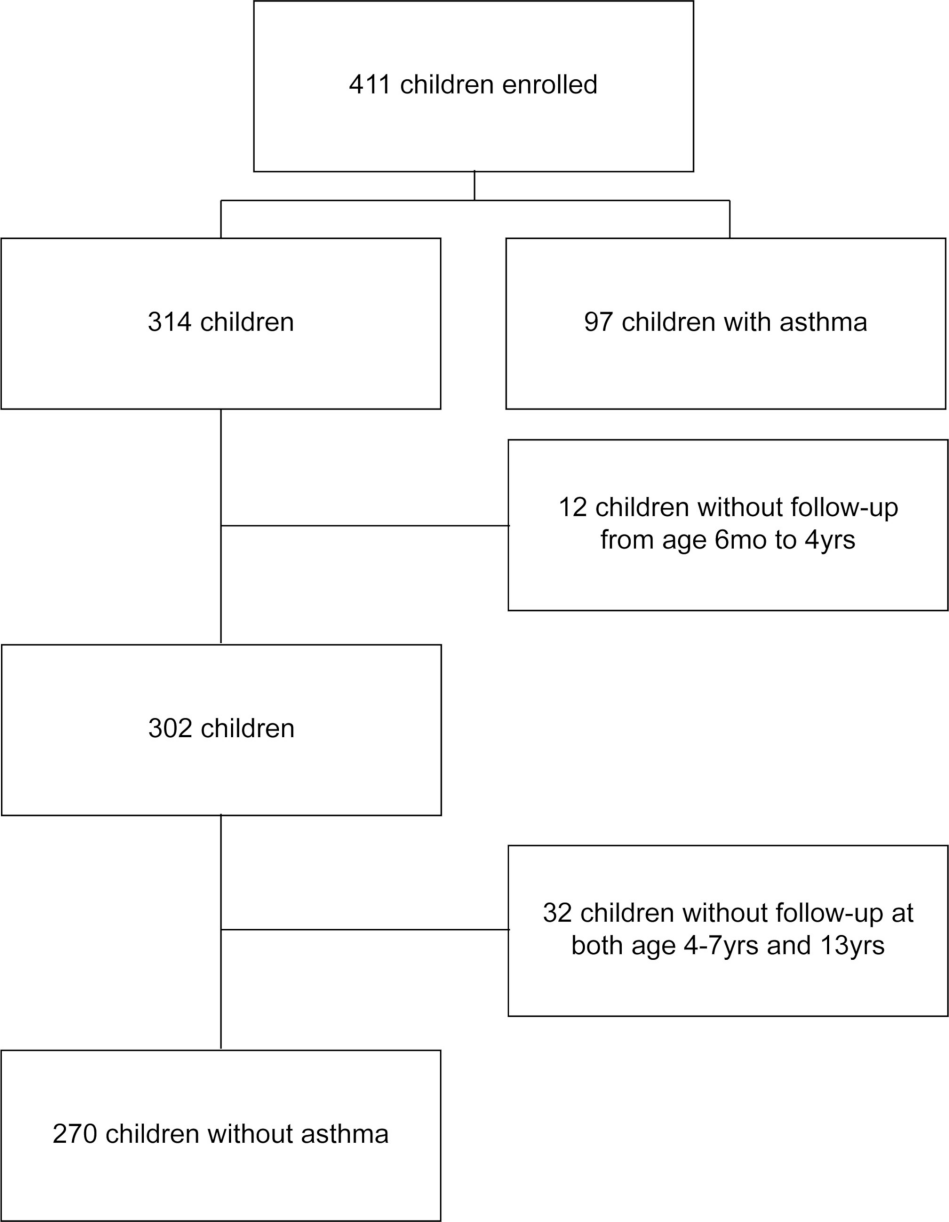
Flowchart of included children.

Of the 411 children enrolled at 1 month of age in COPSAC_2000_, follow-up to age 13 was available for 367 (89%). The number of children ever versus never developing asthma in their first 13 years of life was 97 (27%) versus 270 (73%), respectively (Fig 1). A total of 54 (56%) children remitted before age 13 years; 14 (14%) remitted but relapsed later during childhood. Median age at diagnosis was 2.0 years (IQR 1.2–5.7), median remission age was 6.2 years (IQR 4.2–7.8), and median duration of asthma was 4.0 years (IQR 2.2–5.3). The mean age of the mother at the birth of the child was 30.0 years, and 39% of the children had older siblings. There were no significant differences in baseline characteristics between included and excluded children (Table 1).

**Table 1:** Baseline characteristics.

Characteristic	Included children (*N* = 367)	Excluded children (*N* = 44)	*p*-Value
**Prenatal factors**			
Boys, *n/N* (%)*	182/367 (50%)	21/44 (48%)	0.87
17q21 risk genotype^1^, *n/N* (%)*	103/361 (29%)	11/32 (34%)	0.54
*CDHR3* risk genotype^2^, *n/N* (%)*	106/337 (31%)	7/28 (25%)	0.53
*Filaggrin* risk genotype^3^, *n/N* (%)*	44/363 (12%)	5/32 (16%)	0.57
Paternal asthma, *n/N* (%)*	54/358 (15%)	3/43 (7%)	0.17
Maternal smoking in third trimester, *n/N* (%)*	53/367 (14%)	10/44 (23%)	0.18
**Perinatal factors**			
Gestational age, mean (IQR)**	40 weeks (39; 41)	40 weeks (39; 41)	0.71
Maternal age at birth, mean (IQR)**	30.0 years (26.7; 33.2)	29.8 years (26.1; 33.7)	0.79
Cesarean section, *n/N* (%)*	80/367 (22%)	7/44 (16%)	0.44
Birth weight, mean (IQR)**	3.52 kg (3.20; 3.82)	3.52 kg (3.31; 3.79)	0.93
Birth length, mean (IQR)**	52.3 cm (51.0; 54.0)	52.4 cm (51.8; 53.3)	0.76
**Postnatal factors**			
Social circumstances, mean (SD)**	0.01 (1.00), *N =* 356	−0.19 (0.98), *N =* 26	0.30
Older sibling(s), *n/N* (%)*	139/358 (39%)	12/29 (41%)	0.84
Days of solely breastfeeding, median (IQR)***	122 (90; 155), *N =* 354	110 (14; 132), *N =* 13	0.26
Pets during first year, *n/N* (%)*	92/360 (26%)	12/35 (34%)	0.31
Age at start in daycare, median (IQR)***	12 months (8; 14), *N =* 367	13 months (8; 18), *N =* 34	0.33
Nicotine in hair at 3 years, GM (95% CI)**	0.37 ng (0.31; 0.44), *N =* 309	0.23 ng (0.08; 0.61), *N =* 11	0.31
Weight at 13 years, mean (IQR)**	49.2 kg (42.0; 55.1), *N =* 341	54.2 kg (47.0; 57.2), *N =* 4	0.46
Height at 13 years, mean (IQR)**	160.3 cm (154.3; 165.6), *N =* 341	162.4 cm (155.8; 165.9), *N =* 4	0.68

*Fisher’s exact test.

**Student’s *t* test.

***Wilcoxon 2-sample test.

^1^ Homozygous for the rs7216389 asthma risk allele (T).

^2^Hetero- or homozygous for the rs6967330 asthma risk allele (A).

^3^Mutations in R501X, 2282del4, R2447X, and/or S3247X in the filaggrin coding gene.

GM, geometric mean.

### Lung function development in relation to asthma status

**Fig 2:**
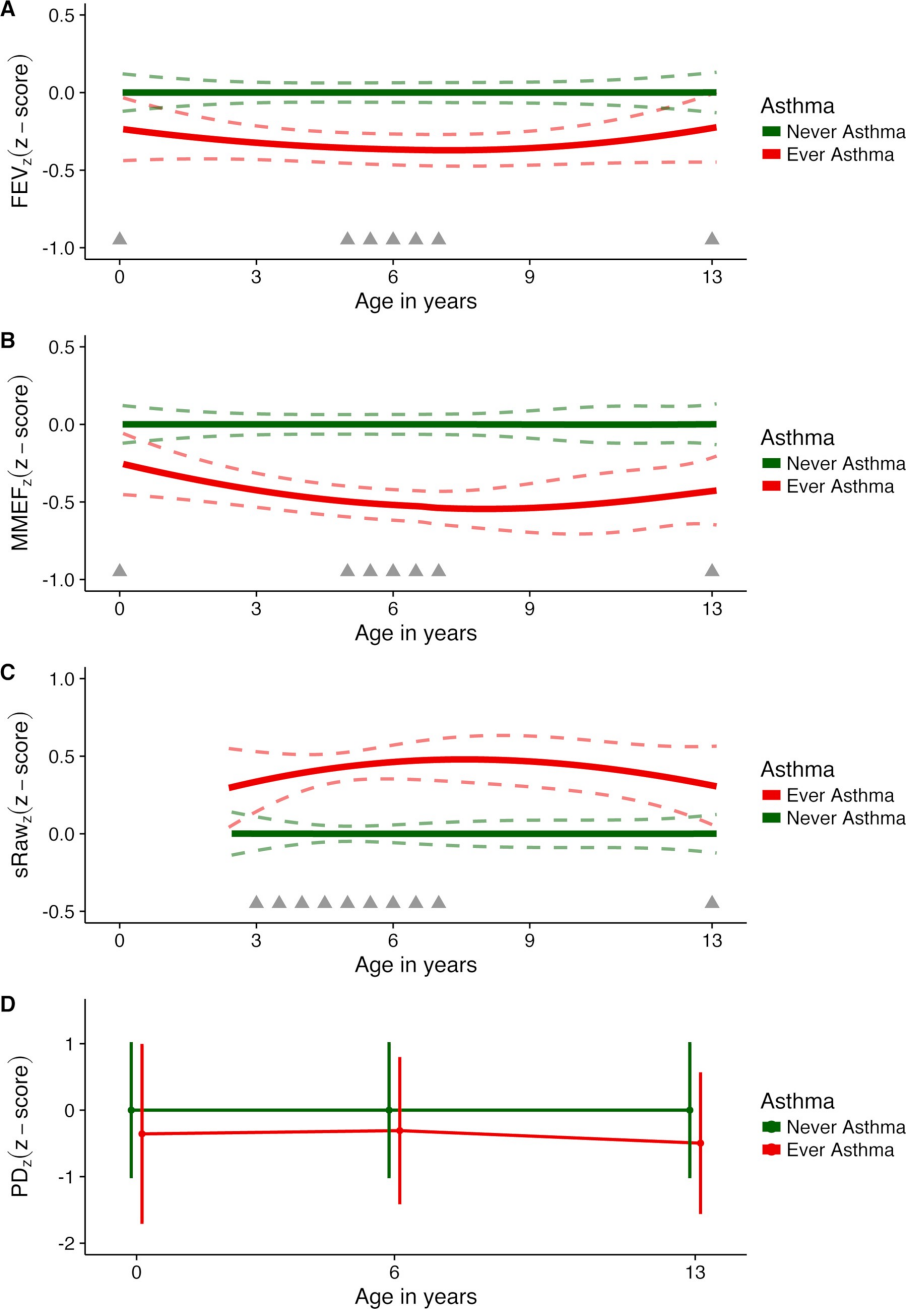
Development of *z-*scored measures of lung function and bronchial reactivity from 1 month to 13 years. *z*-Scores for (A) forced expiratory volume (FEV_z_), (B) maximal mid-expiratory flow (MMEF_z_), (C) specific airway resistance (sRaw_z_), and (D) provocative dose of methacholine (PD_z_). Dashed lines represent 95% confidence intervals; grey triangles indicate timing of measurements. The vertical bars in (D) indicate the exact distribution in terms of age of sampling of the raw data.

The development of FEV_z_ from age 1 month to 13 years in children ever versus never diagnosed with asthma in this period is depicted in Fig 2A, illustrating that children developing asthma already had reduced FEV_z_ as neonates, which persisted until age 13 years as a stable trait without progression or attenuation during childhood (*z-*score difference, −0.31 [95% CI −0.47; −0.15], *p <* 0.001). The development of MMEF_z_ was similar to that of FEV_z_ (Fig 2B) (*z-*score difference, −0.44 [95% CI −0.60; −0.27], *p <* 0.001). sRaw_z_ measurements from age 3 to 13 years showed increased sRaw from age 3 years among children ever versus never developing asthma, which was sustained till age 13 years without progression or attenuation (Fig 2C) (*z-*score difference, +0.40 [95% CI +0.24; +0.56], *p <* 0.001). Finally, bronchial reactivity to methacholine (PD_z_) was persistently increased from age 1 month until age 13 years in children ever versus never diagnosed with asthma in that period (Fig 2D) (i.e., reduced PD_z_; *z-*score difference, −0.40 [95% CI −0.58; −0.22], *p <* 0.001) (Table 2). A sensitivity analysis utilizing the raw, untransformed data showed similar results (Table A in S1 Text). Furthermore, transforming the data led to more normalized residuals (Fig A in S1 Text). Finally, analyses using multiple imputation also showed the same differences in lung function development in children ever versus never diagnosed with asthma; although the effect estimates were slightly attenuated, the results remained significant (Table B in S1 Text).

**Table 2:** Lung function and bronchial reactivity in relation to asthma at age 1 month to 13 years.

Measure	Asthma	*N*	Mean	Difference (95% CI)	*p*-Value
**FEV**					
FEV_z_ *z-*score^*#*^	Never	270	0.00	Reference	**<0.001**
	Ever	97	−0.33	−0.31 (−0.47; −0.15)	
FEV_z_ *z-*score after β_2_-agonist^*#*^	Never	261	0.00	Reference	0.07
	Ever	94	−0.20	−0.16 (−0.34; +0.01)	
Percent change in FEV_1_ after β_2_-agonist^*#*^	Never	261	+3%	Reference	**<0.001**
	Ever	94	+6%	+3% (+2%; +4%)	
Percent change in FEV_1_ after cold dry air^*##*^	Never	182	−3%	Reference	**0.007**
	Ever	69	−7%	−4% (−7%; −1%)	
Percent change in FEV_1_ after exercise^*##*^	Never	210	−8%	Reference	**0.02**
	Ever	78	−12%	−4% (−7%; −1%)	
**MMEF**					
MMEF_z_ *z-*score^*#*^	Never	269	0.00	Reference	**<0.001**
	Ever	97	−0.46	−0.44 (−0.60; −0.27)	
MMEF_z_ *z-*score after β_2_-agonist^*#*^	Never	261	0.00	Reference	**<0.001**
	Ever	94	−0.38	−0.35 (−0.54; −0.16)	
Percent change in MMEF after β_2_-agonist^*#*^	Never	261	+21%	Reference	**0.04**
	Ever	94	+28%	+6% (0%; +12%)	
Percent change in MMEF after cold dry air^*##*^	Never	178	−4%	Reference	**0.03**
	Ever	68	−12%	−8% (−15%; −1%)	
**sRaw**					
sRaw_z_ *z-*score^*#*^	Never	263	0.00	Reference	**<0.001**
	Ever	94	+0.41	+0.40 (+0.24; +0.56)	
sRaw_z_ *z-*score after β_2_-agonist^*#*^	Never	261	0.00	Reference	**0.007**
	Ever	93	+0.25	+0.22 (+0.06; +0.38)	
Percent change in sRaw after β_2_-agonist^*#*^	Never	261	−19%	Reference	**<0.001**
	Ever	93	−23%	−4% (−6%; −2%)	
Percent change in sRaw after cold dry air^*#*^	Never	215	+8%	Reference	**<0.001**
	Ever	76	+16%	+9% (+5%; +13%)	
**PD**					
PD_z_ *z-*score^*#*^	Never	267	0.00	Reference	**<0.001**
	Ever	95	−0.39	−0.40 (−0.58; −0.22)	

Significant *p*-values in bold.

^#^Mixed models.

^##^Student’s *t* test.

FEV, forced expiratory volume; MMEF, maximal mid-expiratory flow; PD, provocative dose of methacholine; sRaw, specific airway resistance.

In order to test whether the relationship between lung function development and asthma status varied with age, we introduced the interaction term asthma status × age at measurement in the models. This analysis showed that there was no interaction between asthma status and age for lung function measurement (Table C in S1 Text), suggesting that these are predetermined traits established in early life and stable throughout childhood. An ancillary analysis of neonatal FEV_0.5_, FEF_50_, and PD_15_ showed significantly decreased forced flows and increased reactivity to methacholine in neonates developing asthma within their first 13 years of life compared to neonates never diagnosed with asthma (Table D in S1 Text).

Children who developed asthma during the first 13 years of life compared to children never developing asthma also had increased bronchodilator response (absolute difference: FEV_1_, +3% [95% CI +2%; +4%], *p <* 0.001; MMEF, +6% [95% CI 0%; 12%], *p =* 0.04; sRaw, −4% [95% CI −6%; −2%], *p <* 0.001), reduced post-bronchodilator *z-*scores for FEV_1_ (−0.16 [95% CI −0.34; 0.01], *p =* 0.07) and MMEF (−0.35 [95% CI −0.54; −0.16], *p <* 0.001), and increased post-bronchodilator *z-*score for sRaw (+0.22 [95% CI +0.06; +0.38], *p =* 0.007).

Reactivity to cold dry air was increased among children ever versus never having asthma (absolute difference: FEV_1_, −4% [95% CI −7%; −1%], *p =* 0.007; MMEF, −8% [95% CI −15%; −1%], *p =* 0.03; and sRaw, +9% [95% CI +5%; +13%], *p <* 0.001). Additionally, children ever versus never diagnosed with asthma experienced a significantly larger drop in FEV_1_ after exercise challenge at age 7 years (absolute difference: FEV_1_, −4% [95% CI −7%; −1%], *p =* 0.02) (Table 2).

### Lung function development in relation to debut, duration, and remission of asthma

Children who developed asthma showed significantly increased airway obstruction and bronchial reactivity before symptom debut compared to children never developing asthma (FEV_z_, −0.29 [95% CI −0.49; −0.09], *p =* 0.004; MMEF_z_, −0.40 [95% CI −0.60; −0.20], *p <* 0.001; sRaw_z_, +0.33 [95% CI +0.10; +0.57], *p =* 0.006; and PD_z_, −0.35 [95% CI −0.59; −0.12], *p =* 0.003).

The airway obstruction and increased bronchial reactivity in children developing asthma were independent of disease duration (change per year: FEV_z_, −0.01 [95% CI −0.07; +0.06], *p =* 0.82; MMEF_z_, −0.01 [95% CI −0.05; +0.03], *p =* 0.68; sRaw_z_, −0.02 [95% CI −0.06; +0.03], *p =* 0.43; and PD_z_, −0.04 [95% CI −0.10; +0.03], *p =* 0.27). Furthermore, the airway obstruction and increased bronchial reactivity did not attenuate after remission (change per year: FEV_z_, +0.02 [95% CI −0.03; +0.06], *p =* 0.44; MMEF_z_, −0.00 [95% CI −0.04; +0.04], *p =* 0.98; sRaw_z_, +0.01 [95% CI −0.03; +0.06], *p =* 0.54; and PD_z_, +0.00 [95% CI −0.07; +0.08], *p =* 0.92) (Table 3).

**Table 3:** Lung function development in relation to asthma debut, duration, and remission.

Measure	*N*	Difference (95% CI)	*p*-Value
***z-*Score before diagnosis relative to children never developing asthma**			
FEV_z_ before diagnosis^*#*^	93	−0.29 (−0.49; −0.09)	**0.004**
MMEF_z_ before diagnosis^*#*^	93	−0.40 (−0.60; −0.20)	**<0.001**
sRaw_z_ before diagnosis^*#*^	32	+0.33 (+0.10; +0.57)	**0.006**
PD_z_ before diagnosis^*#*^	87	−0.35 (−0.59; −0.12)	**0.003**
**Change in *z-*score per year duration of asthma**			
FEV_z_ development^*#*^	92	+0.02 (−0.02; +0.07)	0.36
MMEF_z_ development^*#*^	92	−0.01 (−0.05; +0.03)	0.68
sRaw_z_ development^*#*^	89	−0.02 (−0.06; +0.03)	0.43
PD_z_ development^*#*^	87	−0.04 (−0.10; +0.03)	0.27
**Change in *z-*score per year after remission of asthma**			
FEV_z_ development^*#*^	54	+0.02 (−0.03; +0.06)	0.44
MMEF_z_ development^*#*^	54	−0.00 (−0.04; +0.04)	0.98
sRaw_z_ development^*#*^	51	+0.01 (−0.03; +0.06)	0.54
PD_z_ development^*#*^	48	+0.00 (−0.07; +0.08)	0.92

Significant *p*-values in bold.

^#^Mixed models.

FEV, forced expiratory volume; MMEF, maximal mid-expiratory flow; PD, provocative dose of methacholine; sRaw, specific airway resistance.

### Lung function development in relation to transient versus persistent asthmatic symptoms

Among the 97 children who fulfilled the diagnostic criteria for asthma, 54 children had a transient phenotype and remitted before age 13 years, whereas the remaining 43 children had persistent asthmatic symptoms requiring continued ICS by age 13 years. Lung function development in children with transient asthmatic symptoms compared to children never diagnosed with asthma showed reduced FEV_z_ (*z-*score difference, −0.30 [95% CI −0.51; −0.08], *p =* 0.008) and reduced MMEF_Z_ (−0.36 [95% CI −0.59; −0.14], *p =* 0.002) from age 1 month to 13 years, and increased sRaw_z_ from age 3 years to 13 years (+0.27 [95% CI +0.06; +0.48], *p =* 0.01). PD_z_ was reduced among transiently asthmatic versus healthy children, i.e., indicating increased airway reactivity, but this difference was not significant (−0.15 [95% CI −0.39; +0.09], *p =* 0.23).

**Fig 3:**
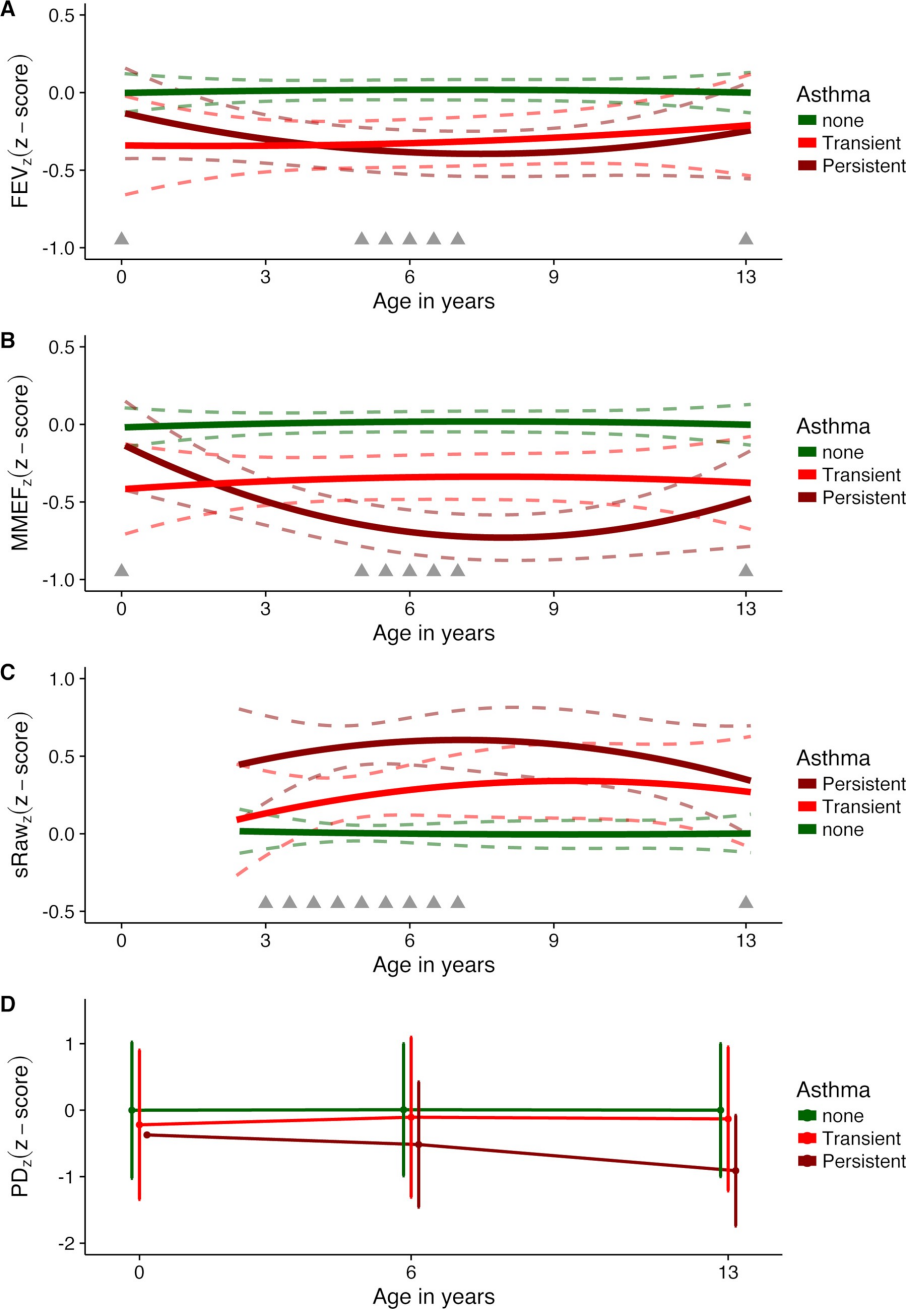
Development of *z-*scored measures of lung function and bronchial reactivity from 1 month to 13 years in children with transient asthma, children with persistent asthma, and children never developing asthma. *z*-Scores for (A) forced expiratory volume (FEV_z_), (B) maximal mid-expiratory flow (MMEF_z_), (C) specific airway resistance (sRaw_z_), and (D) provocative dose of methacholine (PD_z_). Dashed lines represent 95% confidence intervals; grey triangles indicate timing of measurements.

There were no differences in development of FEV_z_, MMEF_z_, or sRaw_z_ among children with transient versus persistent asthmatic symptoms, whereas children with transient compared to persistent symptoms had less airway reactivity to methacholine, i.e., higher PD_z_ (+0.48 [95% CI +0.15; +0.80], *p =* 0.004) (Fig 3; Table E in S1 Text).

### Lung function development in relation to allergic sensitization

At age 13 years, 157 (52%) of 304 tested children had allergic sensitization towards 1 or more of the tested allergens by either a positive skin prick test and/or elevated blood level of specific IgE. Sensitization was diagnosed in 109 (49%) of 223 children never developing asthma, 15 (36%) of 42 children with transient asthmatic symptoms, and in 33 (85%) of 39 children with persistent asthmatic symptoms. Thus, sensitization was more prevalent in children with persistent asthma versus children never developing asthma (chi-squared test, *p <* 0.001) and in children with persistent versus transient asthmatic symptoms (chi-squared test, *p <* 0.001).

There were no differences in development of FEV_z_, MMEF_z_, or sRaw_z_ among children who developed asthma with versus without allergen-specific sensitization to any of the tested allergens, whereas there was a pattern suggesting increased airway reactivity to methacholine among children with asthma who were sensitized by age 13 years, i.e., lower PD_z_ (−0.34 [95% CI −0.70; +0.02], *p =* 0.06) (Table E in S1 Text). Stratifying the analyses on type of allergen as (1) house dust mite, (2) furred animal (dog, cat, horse), or (3) pollen (birch, grass, mugwort) showed similar results except that children with asthma and sensitization to pollen had significantly lower PD_z_ (Table E in S1 Text).

## Discussion

### Primary findings

Children developing asthma at any time during the first 13 years of life had airway obstruction and increased bronchial reactivity that was present at age 1 month without worsening with increased symptom duration and without improvement after remission. This suggests that these are inherent and stable traits not caused by inflammation during symptomatic periods but rather that predispose the child to develop asthmatic symptoms, exaggerated hyperreactivity, and intermittent airway obstruction.

### Strengths and limitations

The primary strength of this study is the thorough single-center close clinical surveillance of a cohort with a follow-up of 89% till age 13 years. The children were seen for clinical evaluation and lung function assessment at the age of 1 month and thereafter at least half-yearly until age 7 years and again at 13 years. Until age 7 years, the parents filled daily diary cards describing the child’s troublesome lung symptoms, thereby diminishing recall bias [26]. Asthma was diagnosed and treated by the COPSAC pediatricians only according to a predetermined algorithm with a conservative definition based on symptom load and response to and relapse after a standard dose and period of ICS [22], thus avoiding diagnostic heterogeneity. Children with asthma were seen for scheduled 3-monthly visits and for unscheduled acute visits during worsening of symptoms and continued daily symptom diary recording beyond age 7 years if symptoms persisted.

The diagnostic algorithm for asthma, which was based on quantitative symptom assessments verified at scheduled and acute care visits to the research unit and response to ICS treatment, could identify asthmatic children with transient, recurrent viral-induced wheeze and children with a persistent phenotype with or without atopy. This is apparent as approximately half of the children diagnosed according to the algorithm had transient symptoms and remitted before age 13 years. The median age at diagnosis was 2 years, implying that a large proportion of the cases were children whose initial episode of wheezing was related to an infection, but half of these children had ICS-dependent asthma by age 13 years. Importantly, not all children with asthma will respond to ICS initially, and because lack of ICS response cannot exclude asthma, we excluded other chronic lung diseases by means of chest X-ray and a sweat chloride test for cystic fibrosis.

A significant advantage of the study is the longitudinal repetitive lung function assessments performed at 11 time points during childhood by means of spirometry and plethysmography as well as bronchial reactivity to cold dry air, exercise, and methacholine challenge in accordance with recognized guidelines [17–20,27]. We did most measurements in the age range from 3 to 7 years; therefore, the shape of the curves is likely to be influenced by the readings at 1 month and 13 years. Still, the birth cohort design assured that lung function was assessed (1) before the debut of symptoms and asthma diagnosis, (2) repeatedly every 6 months in children with and without asthma, and (3) after remission in children outgrowing their asthma. This allowed us to scrutinize how lung function trajectories develop in children with asthma during childhood and how they are affected by duration and remission of symptoms. Our interaction analyses showed no evidence of age influence on the difference in lung function development in children with versus without asthma, but the low numbers in our cohort may preclude us from detecting subtle differences with age.

It is a strength of our study that neonatal spirometry was performed using the raised volume rapid thoracoabdominal compression technique, providing volume-anchored measurements [10], which makes its results comparable with the forced volumes obtained by spirometry later in childhood. This is an important difference compared to previous studies of neonatal lung function in relation to asthma and lung function development during childhood, which used non-volume-anchored methods [12–14,28]. Assessment of tidal breathing patterns is one such method, which is less sensitive, has high intra-individual variability [29,30], and yields results not associated with volume-anchored measurements later in childhood [13].

One previous study of 95 children investigated airway reactivity from age 1 month throughout childhood, but used a non-volume-anchored assessment of histamine challenge that did not correlate with the FEV_1_-based histamine challenge results at school age, highlighting the same problem of using different approaches to determine neonatal lung function and lung function later in childhood [12,31,32].

The limitations of our study are the relatively small sample size, the limited ethnic variation, and the high-risk nature of the COPSAC_2000_ cohort, which hamper the generalizability of the findings. However, mothers without asthma might be reluctant to have their neonates investigated so thoroughly by lung function testing during sedation. Children of asthmatic mothers are a priori at higher risk of asthma and may experience more severe symptoms and have poorer lung function. However, the children not developing asthma also had asthmatic mothers, and we do not expect the relationship between symptoms and lung function development to be different due to asthma predisposition.

We observed *z-*score differences in lung function trajectories with magnitudes between 0.3 and 0.4 in children who developed asthma compared to children not developing asthma. This overall difference in lung function development is not in the abnormal range, which would be less than −2 z-scores, but it should be kept in mind that we excluded measurements obtained during exacerbations, which may have diminished the overall difference between children with versus without asthma.

### Interpretation

We found that airway obstruction and bronchial hyperreactivity related to asthma are fixed traits from age 1 month to age 13 years, without further deterioration from disease duration or improvement after symptom remission. These findings have important implications for our understanding of the underlying pathology, indicating that the symptomatic phases of childhood asthma are not causing the airway obstruction and bronchial hyperreactivity typical of asthma. Instead, we propose that airway obstruction and bronchial hyperreactivity are inherent traits that increase the risk of developing airway inflammation and asthmatic symptoms during childhood. This may explain the lack of effect from early intervention with ICS, which was unable to change the natural course of asthma with respect to both symptom burden and lung function development [2–6].

Our findings are consistent with a follow-up study of children diagnosed with asthma at age 9 years showing that individuals still experiencing symptoms at age 26 years had stable airway obstruction and bronchial reactivity without any progression from the assessments at age 9 years [33]. In addition, a 22-year follow-up of a cohort of 40-year-old adults also showed that reduced lung function at cohort inception remained stable over time [34]. Together with the present study, this suggests that airflow obstruction and bronchial hyperreactivity are inherent and stable deficits measurable already at age 1 month. Thus, even though lung function fluctuates with symptom load and anti-inflammatory treatment over time [35], the lung function trajectory is a static characteristic that might contribute to asthmatic symptoms, exaggerated bronchial reactivity, and intermittent airway obstruction.

Our findings contrast with those of a recent study investigating the development of lung function in children with asthma into adulthood [36], which suggested that the majority of children with asthma (75%) had an abnormal pattern of reduced growth and/or early decline in lung function, i.e., not a stable trait. This difference may be due to the fact that the study only enrolled 5- to 12-year-old children with uncontrolled chronic asthma (symptoms >2 days/week) and severe bronchial hyperreactivity at baseline (>20% drop in FEV_1_ after methacholine challenge), resembling a minority population of the most severely affected children. Even more important, the participants with reduced growth in lung function already had significantly lower lung function at enrollment, suggesting that their lung function trajectories may be a static characteristic established in early childhood. In line with our findings, another recent study demonstrated increased airway resistance among children with persistent wheeze, which tracked from early to late childhood [37].

We suggest that the propensity to develop asthmatic symptoms due to intrinsic and extrinsic factors is increased by underlying inherent airflow obstruction and bronchial hyperreactivity, which are stable traits without progression due to triggering factors or increased symptom duration. On the other hand, we and other groups have shown that allergic sensitization after the age of 6 years, but not earlier in childhood, is associated with an increased risk of developing asthma [38,39], with different risks for different allergens [40]. This raises the possibility that development of allergy may have a deteriorating effect on the lung function trajectory. However, our data in general argue against such a hypothesis as development of airflow obstruction as measured by FEV_z_, MMEF_z_, and sRaw_z_ among children with asthma was independent of allergic sensitization at age 13 years, whilst there was a non-significant suggestion of increased airway reactivity among sensitized asthmatic individuals that was significant in the subgroup with sensitization to pollens, which were the most common allergens in our cohort. These findings are in line with a study of 1,719 15-year-old participants in the German GINIplus birth cohort, which did not show any associations between sensitization and spirometric indices in children with asthma [41], but are in contrast to the American TENOR study of 1,261 children aged 6 to 17 years with severe or difficult-to-treat asthma, which showed an association between increased airflow limitation and higher IgE levels [42]. Importantly, both these studies were cross-sectional analyses; a longitudinal study showed that development of lung function from age 1 month until 11 years was unaffected by atopy [13].

Interestingly, we found that all children who fulfilled the diagnostic criteria for asthma compared to children never diagnosed with asthma had increased airway obstruction, i.e., reduced FEV_z_ and MMEF_z_ (age 1 month to 13 years) and increased sRaw_z_ (age 3 years to 13 years), irrespective of remission of symptoms or not. Thus, fixed airway obstruction was apparent in children with both a transient and a persistent phenotype and without differences in development of FEV_z_, MMEF_z_, and sRaw_z_ between the phenotypes, whereas bronchial reactivity to methacholine (PD_z_) was more pronounced in children with persistent as opposed to transient symptoms. This suggests that airway reactivity in children with transient asthmatic symptoms declines with airway growth through childhood, whereas reduced forced flows and increased airway resistance are fixed traits among children with the transient phenotype, irrespective of airway growth. These findings align with findings from longitudinal lung function measurements from birth until age 16 years in the Tucson Children’s Respiratory Study [43] and from age 3 years until age 11 years in the Manchester Asthma and Allergy Study [37], showing that children with persistent wheeze compared to never wheeze had increased and fixed airway obstruction, which was also apparent for children with early-transient viral-induced wheeze even though they outgrew their symptoms. Furthermore, a smaller Australian study from Perth also showed a suggestion of reduced lung function from birth until age 11 years in children with persistent wheeze, whereas no reduction of lung function was observed for other phenotypes of wheeze [12]. Overall, these studies together with our study suggest a common underlying lung function deficit driving early-transient, typically viral-induced symptoms and persistent symptoms, typically triggered by allergens, pollutants, or exercise.

### Conclusion

In this study we observed that airway obstruction and increased bronchial reactivity associated with childhood asthma were established already at age 1 month, without further deterioration into early adolescence and without relation to asthma symptom duration or remission. This suggests that these chronic deficits are not the result of childhood asthma, but may instead contribute to asthma pathogenesis by increasing the risk of symptoms, exaggerated hyperreactivity, and airway obstruction, implying that preventive measures for improved lung health should focus on the pre- or perinatal period.

## Supporting information

S1 STROBE Checklist Checklist according to the Strengthening the Reporting of Observational Studies in Epidemiology (STROBE) guidelines. (DOC)

S1 Data Data utilized for the analyses presented in the paper. (XLSX)

S1 Text Supplementary text, tables, and figures. (DOCX)

## Abbreviations

COPSAC_2000_: Copenhagen Prospective Studies on Asthma in Childhood 2000
FEF_50_: forced expiratory flow at 50% of expiration
FEV_0.5_: forced expiratory volume in the first 0.5 seconds
FEV_1_: forced expiratory volume in the first second
ICS: inhaled corticosteroid
MMEF: maximal mid-expiratory flow
PD_15_: provocative dose of methacholine producing a 15% decrease in transcutaneous oxygen pressure
PD_20_: provocative dose of methacholine producing a 20% decrease in forced expiratory volume in the first second
PtcO_2_: transcutaneous oxygen pressure
sRaw: specific airway resistance
