# Supplementary material for: Airway obstruction and bronchial reactivity from age 1 month until 13 years in children with asthma: A prospective birth cohort study
Source: PLoS Med. 2019 Jan 8;16(1):e1002722. doi: 10.1371/journal.pmed.1002722 (PMC6324782; doi:10.1371/journal.pmed.1002722)
Supplement: S1 STROBE Checklist — (DOC) [file pmed.1002722.s001.doc]

STROBE Statement—Checklist of items that should be included in reports of ***cohort studies***

|  | Item No | Recommendation |
| --- | --- | --- |
| **Title and abstract** | 1 | (*a*) Indicate the study’s design with a commonly used term in the title or the abstract  *Title: Development of Airway Obstruction and Bronchial Reactivity from age 1 month till 13 years in Children with Asthma: A Prospective Birth Cohort Study* |
|  |  | (*b*) Provide in the abstract an informative and balanced summary of what was done and what was found  *See abstract* |
| Introduction | | |
| Background/rationale | 2 | Explain the scientific background and rationale for the investigation being reported  *Lung function development in childhood is only sparsely investigated and only few cohorts have data from birth. Findings hitherto have suggested that neonatal lung function is reduced in children who later develop asthma, but conclusions have not been consistent. Moreover, it is unknown how lung function develops during asthma symptoms and after asthma symptoms cease. A deeper insight could shear light on some basic mechanisms and pathology regarding childhood asthma.* |
| Objectives | 3 | State specific objectives, including any prespecified hypotheses  *We hypothesized, that reduced airway caliber and bronchial hyperreactivity are inherent and stable traits, which increase the risk of asthmatic symptoms, exaggerated hyperreactivity and intermittent airway obstruction from a superimposed inflammatory process.*  *Objectives: To study lung function development in children with asthma before onset, during and after cessation of asthma symptoms.* |
| Methods | | |
| Study design | 4 | Present key elements of study design early in the paper  *See methods section* |
| Setting | 5 | Describe the setting, locations, and relevant dates, including periods of recruitment, exposure, follow-up, and data collection  *The study was nested in Copenhagen Prospective Studies of Asthma in Childhood 2000 (COPSAC_2000_): a Danish prospective birth cohort study of 411 infants born during 1998-2001 to mothers with a history of asthma. At enrolment at age 1 month, we excluded any child with symptoms of lower airway infection or neonatal mechanical ventilation prior to inclusion, gestational age <36 weeks, and any congenital abnormality or systemic illness. The children were examined at half-yearly scheduled visits until age 7 years, and again at age 13 years, including assessments of lung function eleven times during childhood.* |
| Participants | 6 | (*a*) Give the eligibility criteria, and the sources and methods of selection of participants. Describe methods of follow-up  *Women from the Danish National Birth Cohort Study of approximately 100,000 pregnant women and pregnant women who were attending prenatal clinics were invited to receive further information about the COPSAC_2000_. Women from the greater Copenhagen area who indicated an interest in the study were interviewed by telephone regarding eligibility criteria, including fluency in Danish, a physician’s diagnosis of asthma after the age of 7 years, and a history of daily treatment with inhaled β_2_-agonists or glucocorticoids for a minimum of 2 weeks during 2 seasons or continuously for 1 year.*  *Children attended the clinical research unit at enrollment at 1 month of age, every 6 months until the age of 7 years and at 13 years. Children with acute troublesome lung symptoms were seen by physicians at the clinic.* |
|  |  | (*b*) For matched studies, give matching criteria and number of exposed and unexposed |
| Variables | 7 | Clearly define all outcomes, exposures, predictors, potential confounders, and effect modifiers. Give diagnostic criteria, if applicable  *See description in methods section* |
| Data sources/ measurement | 8* | For each variable of interest, give sources of data and details of methods of assessment (measurement). Describe comparability of assessment methods if there is more than one group  *See description in methods section. Comparability of neonatal lung function measurements to measurements later in childhood is enabled by z-score transformations.* |
| Bias | 9 | Describe any efforts to address potential sources of bias  *We evaluated difference in baseline characteristics between participants and non-participants to address whether the participants were representative of the entire cohort or represented a selected group, which was not the case.* |
| Study size | 10 | Explain how the study size was arrived at  *Of 1,476 pregnant women reporting a history of asthma and interest in the study, 798 (54%) were considered asthmatic after a telephone interview. Of these 798 women, 548 visited the clinical research unit to receive detailed information, and mothers of 452 infants consented to participation. Before enrollment, 2 infants were excluded owing to congenital diseases (heart disease and microcephalia), and 39 infants failed to attend the enrollment visit 1 month after birth. Thus, 411 infants of 394 mothers (9 pairs of twins and 8 siblings) were enrolled in the cohort.* |
| Quantitative variables | 11 | Explain how quantitative variables were handled in the analyses. If applicable, describe which groupings were chosen and why  *Lung function measurements were used as continuous variables. Variables used as baseline characteristics in table 1 are described in online supplement.* |
| Statistical methods | 12 | (*a*) Describe all statistical methods, including those used to control for confounding  *Methods of lung function measurements with repeated measurements were analysed with mixed models with fixed slope and random intercepts for each child. Estimates extracted from the analyses were the fixed effects of groups. To evaluate if lung function changed during asthma symptoms or after remission of asthma symptoms correlation analyses were conducted to examine if time correlated with lung function, i.e. whether lung function changed over time with increasing disease length or time since remission.*  *Methods of lung function measurements assessed only once during childhood were analysed with student’s t-test.* |
|  |  | (*b*) Describe any methods used to examine subgroups and interactions  *As post-hoc subgroup analyses, we investigated development of lung function in children with remission of asthma, i.e. early-transient symptoms, compared to children with persistent symptoms and children never developing asthma. We also investigated development of lung function in children with asthma, who had specific allergen sensitization at age 13 compared to children with asthma without sensitization. The analyses were done with mixed models, evaluating the fixed effect of wheeze phenotype and sensitization status.*  *Interaction analyses of lung function and time were conducted as additional analyses to further exclude that lung function developed differently in children with vs. without asthma during childhood. These are only presented in online supplement.* |
|  |  | (*c*) Explain how missing data were addressed  *Children who were seen at the clinic early in life (0-4yrs) when lung symptoms were most prevalent and late in life (5-13yrs) to ensure correct classification as never asthma and children who at some point fulfilled the asthma criteria were included in the analyses. All quality controlled lung function measurements from these children were included in the analyses. The mixed models used to examine the longitudinal measurements took account of missing data as the individual child was assigned as repeated subject in the models.* |
|  |  | (*d*) If applicable, explain how loss to follow-up was addressed  *Children without asthma diagnosis not attending at least one visit from age 6 months to 4 years and at least one visit after age 4 years were excluded from analysis as data were found insufficient to fully conclude that asthma criteria were not fulfilled. A drop-out analysis is included as table 1 in the study.* |
|  |  | (*e*) Describe any sensitivity analyses  *A sensitivity analysis utilizing raw, untransformed data showed similar results as the normalized data. This is included as an online figure.* |
| Results | | |
| Participants | 13* | (a) Report numbers of individuals at each stage of study—eg numbers potentially eligible, examined for eligibility, confirmed eligible, included in the study, completing follow-up, and analysed  *411 children enrolled, of these 97 children fulfilled the asthma criteria at some point in childhood and were included in the study. Of the remaining 314 children, 12 were not seen at visits from 6 months to 4 years of age and 32 were not seen at visits from age 4 years to 13 years, leaving 270 children without asthma to be included in the analyses.* |
|  |  | (b) Give reasons for non-participation at each stage  *Non-participation was due to lack of participation at scheduled visits.* |
|  |  | (c) Consider use of a flow diagram  *Included as figure 1* |
| Descriptive data | 14* | (a) Give characteristics of study participants (eg demographic, clinical, social) and information on exposures and potential confounders  *See baseline characteristics in table 1* |
|  |  | (b) Indicate number of participants with missing data for each variable of interest  *Indicated as N for each variable in the tables* |
|  |  | (c) Summarise follow-up time (eg, average and total amount)  *Of the included children: 204 attended all 17 scheduled visits, of these 64 had asthma ever; 52 attended 16 visits, of these 14 had asthma ever; 17 attended 15 visits, of these 3 had asthma ever; 17 attended 14 visits, of these 2 had asthma ever; 11 attended 13 visits, of these 4 had asthma ever; 10 attended 12 visits, of these 2 had asthma ever; 7 attended 11 visits, of these 0 had asthma ever; 15 attended 10 visits, of these 2 had asthma ever; 5 attended 9 visits, of these 0 had asthma ever; 8 attended 8 visits, of these 2 had asthma ever; 7 attended 7 visits, of these 1 had asthma ever; 7 attended 6 visits, of these 1 had asthma ever; 4 attended 5 visits, of these 2 had asthma ever; 3 attended 4 visits, of these 0 had asthma ever.* |
| Outcome data | 15* | Report numbers of outcome events or summary measures over time  *See results section* |
| Main results | 16 | (*a*) Give unadjusted estimates and, if applicable, confounder-adjusted estimates and their precision (eg, 95% confidence interval). Make clear which confounders were adjusted for and why they were included  *Only adjusted analyses were of lung function during asthma diagnosis, which were adjusted for time since remission as some of the children were in remission of asthma symptoms, yet did they still have a previous duration of asthma to be analysed. A table showing crude and adjusted estimates with 95% confidence intervals is included in online supplement.* |
|  |  | (*b*) Report category boundaries when continuous variables were categorized  *No continuous variables were categorized* |
|  |  | (*c*) If relevant, consider translating estimates of relative risk into absolute risk for a meaningful time period  *No results were reported as relative risk* |
| Other analyses | 17 | Report other analyses done—eg analyses of subgroups and interactions, and sensitivity analyses  *All analyses are reported in the article or supplement material* |
| Discussion | | |
| Key results | 18 | Summarise key results with reference to study objectives  *Children developing asthma at any time during the first 13 years of life had airway obstruction and increased bronchial reactivity, which was present at age 4 weeks without worsening with increased symptom duration or improvement after remission. This suggests that these are inherent and stable traits not caused by inflammation during symptomatic periods but rather predisposing the child to develop asthmatic symptoms, exaggerated hyperreactivity and intermittent airway obstruction.* |
| Limitations | 19 | Discuss limitations of the study, taking into account sources of potential bias or imprecision. Discuss both direction and magnitude of any potential bias  *See discussion section* |
| Interpretation | 20 | Give a cautious overall interpretation of results considering objectives, limitations, multiplicity of analyses, results from similar studies, and other relevant evidence  *We found that airway obstruction and bronchial hyperreactivity related to asthma are fixed traits from age 4 weeks to age 13, without further deterioration from disease duration or improvement after symptom remission. These findings have important implications for our understanding of the underlying pathology, indicating that the symptomatic phases of childhood asthma are not causing the airway obstruction and bronchial hyperreactivity typical of asthma. Instead, we speculate that these are inherent traits increasing the risk of developing asthmatic symptoms, exaggerated hyperreactivity and intermittent airway obstruction from superimposed mucosal inflammation associated with asthma.* |
| Generalisability | 21 | Discuss the generalisability (external validity) of the study results  *The limitation of our study is the high-risk nature of the COPSAC_2000_ cohort, which hampers generalizability of the findings. Moreover as indicated by the high remission rate the cohort contains only few children with severe asthma, which potentially could have another lung function trajectory.* |
| Other information | | |
| Funding | 22 | Give the source of funding and the role of the funders for the present study and, if applicable, for the original study on which the present article is based  *COPSAC is funded by private and public research funds all listed on www.copsac.com. The Lundbeck Foundation (Grant no R16-A1694); The Ministry of Health (Grant no 903516); Danish Council for Strategic Research (Grant no 0603-00280B) and The Capital Region Research Foundation have provided core support for COPSAC. No pharmaceutical company was involved in the study. The funding agencies did not have any role in design and conduct of the study; collection, management, and interpretation of the data; or preparation, review, or approval of the manuscript.* |

*Give information separately for exposed and unexposed groups.

**Note:** An Explanation and Elaboration article discusses each checklist item and gives methodological background and published examples of transparent reporting. The STROBE checklist is best used in conjunction with this article (freely available on the Web sites of PLoS Medicine at http://www.plosmedicine.org/, Annals of Internal Medicine at http://www.annals.org/, and Epidemiology at http://www.epidem.com/). Information on the STROBE Initiative is available at http://www.strobe-statement.org.
