# Supplementary material for: Airway obstruction and bronchial reactivity from age 1 month until 13 years in children with asthma: A prospective birth cohort study
Source: PLoS Med. 2019 Jan 8;16(1):e1002722. doi: 10.1371/journal.pmed.1002722 (PMC6324782; doi:10.1371/journal.pmed.1002722)
Supplement: S1 Text — (DOCX) [file pmed.1002722.s003.docx]

**Supplementary Text, Tables and Figures**

# Methods

Description of covariates

17q21 and *CDHR3* genotype: Genotyping of 17q21 (rs7216389) and *CDHR3* (rs6967330) variants was performed using the Infinium HumanOmniExpressExome BeadChip array, performing sample and marker QC as previously reported^1^.

*Filaggrin* genotype: Genotyping for common loss-of-function mutations in *filaggrin*, R501X, 2282del4, R2447X and S3247X was performed as previously described^2^.

Paternal asthma: Fathers ever diagnosed with asthma by doctor or ever receiving medical treatment for asthma.

Maternal smoking 3rd trimester: Mothers smoking at least one cigarette pr. week on average during third trimester of pregnancy.

Gestational age: Days between first day of last menstruation and birth.

Sectio: Delivery by acute or planned sectio.

Social circumstances: Z-score constructed from the first component of a principal component analysis based on maternal age, maternal level of education and household income when the child was 2 years of age. The first component explains 52% of the variance in the data.

Older siblings: Any older children living at same address during first year of life.

Days of solely breastfeeding: Days the child was breastfed without any other nutrient being part of the diet.

Pets during 1st year: Children exposed to any furred animal on a daily basis during the first year of life.

Age at start in daycare: The age of the child at first day child spent in institution with non-related children.

Nicotine in hair at 3 years: The amount of nicotine found in the hair at 3 years. Detection limit: 0.01ng/mg hair.

Description of multiple imputation:

For the four lung function measurements obtained longitudinal (FEV1, MMEF, sRaw and PD) a proportion of the scheduled observations are missing. In order to investigate the impact of the missing observations, a multiple imputation procedure is employed using two paradigms:

- One based on either the 367 children, where there is full follow-up to 13 years and known asthma status.

- One based on 409 children from which 42 do not have full follow-up with respect to asthma status at 13 years, and hence must be estimated from data. Two children are removed from this latter analysis having zero measures of lung function.

As predictors, the observed measurements of the remaining three lung function measures, as well as the visit age are used. Further, the individual child is used as random factor.

The imputations are completed for the responses individually restricting only missing data on scheduled visits to be imputed.

The 2l.norm model for multilevel imputation of responses from a two-level mixed model with heterogeneous within group (child) variance is used for imputing responses, whereas logistic regression is used for asthma status in the n = 409 imputations.

In comparison, the effect estimates are compared with a model based on all observations from n = 367 children, as well as for a model based on fully compliant children, that is; including only those children who have full follow-up data with respect to lung function measurements.

The mice package (version 3.3.0) for R is used, with a total of n = 100 imputations for each response.

# Results

**Imputation**

For all four longitudinal lung function responses, the imputations show no bias in comparison with the observed data. However, for PD in both imputations schemes, there are both positive and negative outliers inflating the variance of the distribution (results not shown). The effect estimates from the imputed models are shown in **Table B** along with estimates based on observed data (also reported in **Table 2**) and data from fully compliant children.

As expected the results attenuates, when imputing missing observations. However, for all the four lung function responses the difference between asthmatics and non-asthmatics remains significant. Including n = 42 children within unknown asthma status further attenuates the results due to the uncertainty introduced for prediction of asthma status. Restricting the analysis to only include data from children with a full follow-up in terms of lung function does not change the results, however, for sRaw the number of children fulfilling this criterion is very low (n = 4 and n = 29) resulting in high uncertainty.

**Table A:** A comparison of the relative effect estimates (estimate/SE) of diseases status (asthma ever) for models with pre-corrected responses (for sex, height, age and visit) and adjusted (for the same factors).

| **Lung Function** | **Model** | **Relative effect** |
| --- | --- | --- |
| FEV1 | Corrected | -3.35 |
| FEV1 | Adjusted | -2.97 |
| MMEF | Corrected | -4.66 |
| MMEF | Adjusted | -4.39 |
| PD | Corrected | -4.29 |
| PD | Adjusted | -4.28 |
| sRaw | Corrected | 4.95 |
| sRaw | Adjusted | 5.22 |

**Table B:** Effect estimates with 95% CI comparing children with vs. without asthma using lung function dataset with multiple imputation (100 imputations) using n = 367 children with asthma status up to 13 years as well as n = 409 children for which asthma status is also imputed. In comparison, models based on observed data as well as data from fully compliant individuals.

| **Lung function measurements** | **Method** | **No. Observations** | **No. Children** | **No. Missing** | **% Missing** | **Estimate** | **SE** | **CI low** | **CI high** |
| --- | --- | --- | --- | --- | --- | --- | --- | --- | --- |
| FEV_z_ z-score | Observed data | 2046 | 367 | 0 | 0.0 | -0.31 | 0.08 | -0.47 | -0.15 |
| FEV_z_ z-score | Imputed | 2569 | 367 | 523 | 16.9 | -0.28 | 0.08 | -0.45 | -0.11 |
| FEV_z_ z-score | Imputed as many as possible | 2863 | 409 | 766 | 21.1 | -0.20 | 0.08 | -0.36 | -0.03 |
| FEV_z_ z-score | Fully compliant children | 1085 | 155 | 0 | 0.0 | -0.38 | 0.11 | -0.60 | -0.16 |
| % change in FEV_1_ post β_2_-agonist | Observed data | 1230 | 355 | 0 | 0.0 | 0.03 | 0.01 | 0.02 | 0.04 |
| % change in FEV_1_ post β_2_-agonist | Imputed | 1835 | 367 | 605 | 24.8 | 0.02 | 0.01 | 0.01 | 0.04 |
| % change in FEV_1_ post β_2_-agonist | Imputed as many as possible | 1795 | 359 | 558 | 23.7 | 0.02 | 0.01 | 0.01 | 0.04 |
| % change in FEV_1_ post β_2_-agonist | Fully compliant children | 430 | 86 | 0 | 0.0 | 0.04 | 0.01 | 0.01 | 0.07 |
| sRaw_z_ z-score | Observed data | 2375 | 357 | 0 | 0.0 | 0.40 | 0.08 | 0.24 | 0.56 |
| sRaw_z_ z-score | Imputed | 4037 | 367 | 1662 | 29.2 | 0.33 | 0.08 | 0.17 | 0.50 |
| sRaw_z_ z-score | Imputed as many as possible | 4059 | 369 | 1665 | 29.1 | 0.30 | 0.08 | 0.15 | 0.46 |
| sRaw_z_ z-score | Fully compliant children | 44 | 4 | 0 | 0.0 | 0.18 | 0.36 | -0.46 | 0.81 |
| sRaw_z_ z-score | Fully compliant children* | 290 | 29 | 0 | 0.0 | 0.37 | 0.21 | -0.05 | 0.79 |
| PD_z_ z-score | Observed data | 872 | 362 | 0 | 0.0 | -0.40 | 0.09 | -0.58 | -0.22 |
| PD_z_ z-score | Imputed | 1101 | 367 | 229 | 17.2 | -0.34 | 0.12 | -0.58 | -0.10 |
| PD_z_ z-score | Imputed as many as possible | 1203 | 401 | 287 | 19.3 | -0.31 | 0.10 | -0.50 | -0.12 |
| PD_z_ z-score | Fully compliant children | 591 | 197 | 0 | 0.0 | -0.35 | 0.12 | -0.58 | -0.11 |

**Model based on fully compliant children excluding visit at age 2.5 years.*

Table C: Interaction analysis of lung function development in children with and without asthma. The mixed model is: “lung function development” = “asthma status” “asthma status” x “age at measurement”

|  | **Estimate of interaction term [95% CI]** | **p-value** |
| --- | --- | --- |
| FEV_z_ z-score^#^ | -0.00 [-0.02; +0.02] | 0.82 |
| MMEF_z_ z-score^#^ | -0.01 [-0.03; +0.01] | 0.34 |
| sRaw_z_ z-score^#^ | -0.00 [-0.03; +0.02] | 0.42 |
| PD_z_ z-score^#^ | -0.02 [-0.04; +0.01] | 0.58 |

*^#^Mixed models, 95% confidence interval (95%CI).*

Table D: Analysis of neonatal lung function in children who later develop asthma compared to children without asthma

| **Neonatal lung function** | **Asthma** | **N** | **Mean** | **Difference [95%CI]** | **p-value** |
| --- | --- | --- | --- | --- | --- |
| FEV_0.5_^##^ | Never | 268 | 0.0 | reference | 0.03 |
|  | Ever | 93 | -0.25 | -0.25 [-0.47; -0.03] |  |
| FEF_50_^##^ | Never | 266 | 0.0 | reference | 0.02 |
|  | Ever | 93 | -0.27 | -0.27 [-0.48; -0.05] |  |
| PD_15_^##^ | Never | 241 | 0.0 | reference | 0.03 |
|  | Ever | 82 | -0.36 | -0.36 [-0.67; -0.04] |  |

*^##^Student’s t-test, 95% confidence interval (95%CI).*

Table E: Analysis of lung function development from age 4 weeks to 13 years in children with early-transient asthma, persistent asthma and no asthma.

|  | **FEV_z_ difference**  **[95%CI], p** | **MMEF_z_ difference [95%CI], p** | **sRaw_z_ difference [95%CI], p** | **PD_z_ difference [95%CI], p** |
| --- | --- | --- | --- | --- |
| Difference between transient and no asthma^#^ | -0.30 [-0.51; -0.08], p=0.008 | -0.36 [-0.59; -0.14], p=0.002 | +0.27 [+0.06; +0.48], p=0.01 | -0.15 [-0.39; +0.09], p=0.23 |
| Difference between persistent and no asthma^#^ | -0.32 [-0.54; -0.09], p=0.007 | -0.54 [-0.78; -0.30], p<0.001 | +0.49 [+0.27; +0.71], p<0.001 | -0.62 [-0.88; -0.37], p<0.001 |
| Difference between transient and persistent asthma^#^ | +0.02 [-0.27; +0.31], p=0.89 | +0.18 [-0.12; +0.48], p=0.25 | -0.22 [-0.50; +0.06], p=0.12 | +0.48 [+0.15; +0.80], p=0.004 |

*^#^Mixed models, 95% confidence interval (95%CI).*

Table E: Analysis of lung function development from age 4 weeks to 13 years in children with asthma with and without sensitization at 13 years.

| **Lung function measurements** | **Asthma** | **N** | **Mean** | **Difference [95%CI]** | **p-value** |
| --- | --- | --- | --- | --- | --- |
| FEV_z_ z-score^#^ | Without sensitization | 33 | -0.30 | reference | 0.99 |
|  | With sensitization | 48 | -0.35 | -0.00 [-0.30; +0.30] |  |
| FEV_z_ z-score^#^ | Without sensitization | 53 | -0.36 | reference | 0.18 |
|  | With sensitization to HDM | 24 | -0.25 | +0.14 [-0.19; +0.47] |  |
| FEV_z_ z-score^#^ | Without sensitization | 41 | -0.23 | reference | 0.09 |
|  | With sensitization to pollen | 36 | -0.45 | -0.26 [-0.56; +0.05] |  |
| FEV_z_ z-score^#^ | Without sensitization | 52 | -0.28 | reference | 0.33 |
|  | With sensitization to furred animals | 24 | -0.46 | -0.16 [-0.50; +0.17] |  |
| MMEF_z_ z-score^#^ | Without sensitization | 33 | -0.43 | reference | 0.89 |
|  | With sensitization | 48 | -0.49 | -0.02 [-0.33; +0.29] |  |
| MMEF_z_ z-score^#^ | Without sensitization | 53 | -0.52 | reference | 0.14 |
|  | With sensitization to HDM | 24 | -0.31 | +0.25 [-0.08; +0.58] |  |
| MMEF_z_ z-score^#^ | Without sensitization | 41 | -0.39 | reference | 0.36 |
|  | With sensitization to pollen | 36 | -0.53 | -0.14 [-0.45; +0.17] |  |
| MMEF_z_ z-score^#^ | Without sensitization | 52 | -0.45 | reference | 0.87 |
|  | With sensitization to furred animals | 24 | -0.51 | -0.03 [-0.36; +0.31] |  |
| sRaw_z_ z-score^#^ | Without sensitization | 32 | 0.30 | reference | 0.61 |
|  | With sensitization | 48 | 0.45 | +0.08 [-0.25; +0.42] |  |
| sRaw_z_ z-score^#^ | Without sensitization | 53 | 0.36 | reference | 0.94 |
|  | With sensitization to HDM | 24 | 0.47 | -0.02 [-0.39; +0.36] |  |
| sRaw_z_ z-score^#^ | Without sensitization | 40 | 0.28 | reference | 0.32 |
|  | With sensitization to pollen | 36 | 0.48 | +0.17 [-0.17; +0.51] |  |
| sRaw_z_ z-score^#^ | Without sensitization | 51 | 0.37 | reference | 1.00 |
|  | With sensitization to furred animals | 24 | 0.43 | -0.00 [-0.37; +0.37] |  |
| PD_z_ z-score^#^ | Without sensitization | 33 | -0.21 | reference | 0.06 |
|  | With sensitization | 47 | -0.53 | -0.34 [-0.70; +0.02] |  |
| PD_z_ z-score^#^ | Without sensitization | 53 | -0.42 | reference | 0.44 |
|  | With sensitization to HDM | 23 | -0.26 | +0.16 [-0.25; +0.57] |  |
| PD_z_ z-score^#^ | Without sensitization | 40 | -0.15 | reference | **0.009** |
|  | With sensitization to pollen | 36 | -0.63 | -0.48 [-0.84; -0.12] |  |
| PD_z_ z-score^#^ | Without sensitization | 51 | -0.30 | reference | 0.32 |
|  | With sensitization to furred animals | 24 | -0.50 | -0.20 [-0.60; +0.20] |  |

*^#^Mixed models, 95% confidence interval (95%CI). HDM=house dust mites.*

Fig A: Graphical presentation of residuals of lung function measurements before and after transformation.

# References

1. Bønnelykke K, Sleiman P, Nielsen K, *et al.* A genome-wide association study identifies CDHR3 as a susceptibility locus for early childhood asthma with severe exacerbations. *Nat Genet* 2014; **46**: 51–5.

2 . Palmer CNA, Irvine AD, Terron-Kwiatkowski A, *et al.* Common loss-of-function variants of the epidermal barrier protein filaggrin are a major predisposing factor for atopic dermatitis. *Nat Genet* 2006; **38**: 441–6.
